# In Vitro Safety, Off-Target and Bioavailability Profile of the Antiviral Compound Silvestrol

**DOI:** 10.3390/ph15091086

**Published:** 2022-08-31

**Authors:** Susanne Schiffmann, Sandra Gunne, Thomas Ulshöfer, Marina Henke, Luise A. Roser, Ann-Kathrin Schneider, Jindrich Cinatl, Dominique Thomas, Yannick Schreiber, Pia Viktoria Wagner, Arnold Grünweller, Michael J. Parnham

**Affiliations:** 1 Fraunhofer Institute for Translational Medicine and Pharmacology (ITMP), Theodor-Stern-Kai 7, 60596 Frankfurt am Main, Germany; 2 Pharmazentrum Frankfurt/ZAFES, Department of Clinical Pharmacology, Goethe-University Hospital Frankfurt, Theodor-Stern-Kai 7, 60590 Frankfurt am Main, Germany; 3 Institute of Medical Virology, University Hospital Frankfurt, Goethe University, Paul-Ehrlich-Str. 40, 60596 Frankfurt am Main, Germany; 4 Institute of Pharmaceutical Chemistry, Philipps-University Marburg, Marbacher Weg 6, 35032 Marburg, Germany

**Keywords:** silvestrol, rocaglates, antiviral, RNA helicase eIF4A, safety profile, micronuclei assay, cellular uptake assay, transport assay

## Abstract

We characterized the in vitro safety and bioavailability profile of silvestrol, a compound effective against various viruses, such as corona- and Ebolaviruses, with an EC_50_ value of about 5 nM. The cytotoxic profile of silvestrol was assessed in various cancer cell lines, as well as the mutagenic and genotoxic potential with Ames and micronuclei tests, respectively. To identify off-target effects, we investigated whether silvestrol modulates G-protein coupled receptor (GPCR) signaling pathways. To predict the bioavailability of silvestrol, its stability, permeability and cellular uptake were determined. Silvestrol reduced viability in a cell-type-dependent manner, mediated no off-target effects via GPCRs, had no mutagenic potential and minor genotoxic effects at 50 nM. Silvestrol did not disturb cell barrier integrity, showed low membrane permeability, was stable in liver microsomes and exhibited good cellular uptake. Efficient cellular uptake and increased cytotoxicity were observed in cell lines with a low expression level of the transport protein P-glycoprotein, the known efflux transporter of silvestrol. In conclusion, silvestrol showed low permeability but good cellular uptake and high stability. Cell-type-dependent cytotoxicity seems to be caused by the accumulation of silvestrol in cells lacking the ability to expel silvestrol due to low P-glycoprotein levels.

## 1. Introduction

Virus-mediated infectious diseases, such as coronavirus-induced disease 2019 (COVID-19), caused by severe acute respiratory syndrome coronavirus 2 (SARS-CoV-2), are global health challenges. Moreover, the treatment options for virus-mediated diseases are limited, so the development of well-tolerated, as well as efficient, antiviral therapies against emerging and re-emerging viruses is a high priority. Silvestrol could be a promising new broad-spectrum antiviral, since it inhibits the replication of Hepatitis E- [1], Ebola- [2], Zika- [3] Chikungunya- [4], Lassa-[5], Crimean Congo hemorrhagic fever [5], as well as subtypes of Picorna- [6] and coronaviruses [6].

Several viruses need the ATP-dependent DEAD-box RNA helicase elF4A for the translation of their mRNAs; therefore, the inhibition of this host enzyme by silvestrol is an interesting antiviral approach [7,8]. The advantages of targeting host factors include a decreased risk of escape mutations by the virus [9,10]. The disadvantages of such a strategy are possible pleiotropic side effects [11]. However, the inhibition by silvestrol and other rocaglates of the viral 5′ mRNA structure unwinding activity of eIF4A appears to be highly specific, which should reduce the risk of side effects. Since silvestrol was initially described as an anti-cancer drug agent [12,13,14], the assessment of the safety, off-target and pharmacokinetic profile is essential for its preclinical development as a broad-spectrum antiviral.

For the in vitro safety profile viability testing in various cancer cell lines, a micronucleus and an Ames test were performed. For detection of possible off-target effects mediated by GPCR signaling, a cyclic adenosine monophosphate (cAMP), an inositol 1,4,5-triphosphate (IP_3_) and a Ca^2+^-release assay were used. For the in vitro pharmacokinetic profile, a stability assay, a transport assay and a cellular uptake assay were used to gain initial insights into the expected bioavailability of silvestrol [15,16]. Here, these aspects were explored in standard cell culture test systems to assess the druggability of silvestrol.

## 2. Results

### 2.1. Silvestrol Reduced Cell-Type-Specific Viability in Cancer Cells

**Figure 1:**
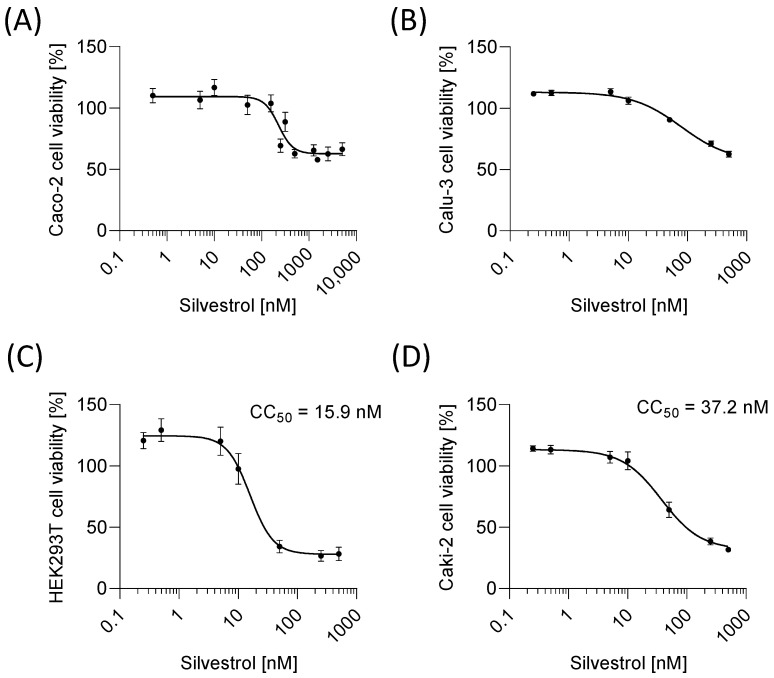
Impact of silvestrol on cell viability. For the cell viability assay, Caco-2 (**A**), Calu-3 (**B**), HEK293T (**C**) and Caki-2 (**D**) cells were incubated with various concentrations of silvestrol over a time period of 48 h. The absorbance of DMSO-treated cells was set to 100% and samples treated with silvestrol were correlated to the absorbance of the respective DMSO value to calculate cell viability. The experiments were performed in biological and technical triplicates. Data are expressed as mean ± SEM. The CC_50_ values were calculated with a non-linear fitting model using GraphPad Prism Version 8.4.3 (GraphPad Software, San Diego, CA, USA) only for HEK293T and Caki-2 cells because in Caco-2 and Calu-3 cells silvestrol did not reduce viability below 50%.

Cytotoxicity testing is a prerequisite for the preclinical characterization of a drug. First, we addressed whether silvestrol affects cell viability and proliferation. Hughes et al. recommended, for non-toxic concentrations, at least 50-fold higher values than the inhibitor concentration (IC)_50_ of the test drug [16]. Since silvestrol has an EC_50_ value (50% reduction of the virus titer) of about 5 nM for MERS-CoV, HCoV-229E [6] and Chikungunya virus [5], we used a compound range from 0.25 to 500 nM. As silvestrol also has potent anti-tumor effects, its cytotoxicity in cancer cell lines and in primary cells has already been investigated. For silvestrol, a CC_50_ of 9.42 nM in the lung cancer cell line A549 [5], of 0.7 nM in the colon cancer cell line HT29 [17], of 30 nM in the hepatocellular carcinoma cell line Huh-7 [6] and of 5 nM in the embryonic kidney cell line HeLa [6] was observed, whereas in the lung fibroblast cell line MRC-5, no cytotoxicity up to 50 µM was detected [5]. In primary human immune cells, a CC_50_ value of 29 nM in primary monocytes, of 46 nM in primary M1 macrophages and no cytotoxic effects up to 100 nM in primary M2 macrophages, T cells and primary dendritic cells were observed [9]. Cell viability was determined with a formazan-based assay in the colon cancer cell line Caco-2, in the lung cancer cell line Calu-3, in the liver cell line HepG2, in the embryonal kidney cell line HEK293T and in the kidney cancer cell line Caki-2. The cytotoxic effect of silvestrol was cell-type dependent. Silvestrol has only minor effects on Caco-2 and HepG2 cells, followed by the lung cancer cell line Calu-3 (Figure 1A,B and Figure S1, Supplementary Materials). Surprisingly, silvestrol showed the strongest anti-proliferative effects in HEK293T cells with a CC_50_ of 15.9 nM (Figure 1C). To investigate whether kidney cells are particularly sensitive to silvestrol, Caki-2 as a further kidney cell line was evaluated. Silvestrol revealed a CC_50_ value of 37.2 nM in Caki-2 cells (Figure 1D). These data and literature data (see above) indicate that the anti-proliferative effect of silvestrol is, indeed, cell-type dependent.

### 2.2. Silvestrol Has No Mutagenic and Low Genotoxic Potential

**Figure 2:**
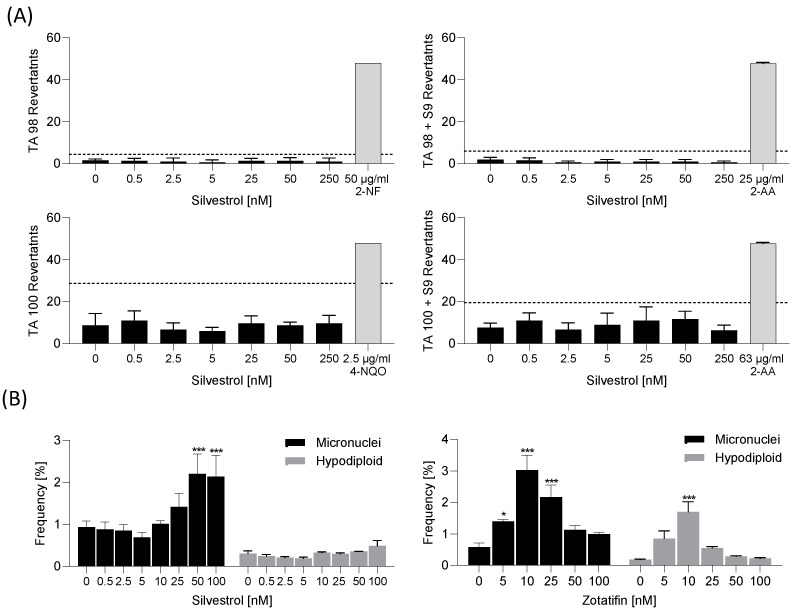
Mutagenic potential of silvestrol. (**A**) The Ames test was conducted with two *Salmonella typhimurium* strains TA98 and TA100 in the presence or absence of liver homogenate S9 to simulate putative metabolic conversions of silvestrol by liver enzymes. The Ames MPF 98/100 assay from Xenometrix was performed as described by the supplier. The Ames MPF calculation sheet provided by Xenometrix was used for data analysis. The experiment was performed once in triplicate as suggested by the supplier. The dashed line indicates 2-fold increase over baseline. Data are expressed as mean ± SD. Abb. 2-AA, 2-aminoanthracene; 2-NF, 2-nitrofluorene; 4-NQO, 4-nitroquinoline-N-oxide. (**B**) For the micronuclei assay, HepG2 cells were incubated with increasing concentrations of silvestrol and zotatifin or vehicle for 44 h. The analysis of micronuclei and hypodiploid cells was conducted by flow cytometry as recommended by the supplier. The experiments were performed in biological triplicates. Data are expressed as mean ± SEM. For statistical analysis, two-way ANOVA with Dunnett’s multiple comparisons test was used. * *p* < 0.05, *** *p* < 0.001 indicate significant differences between compound samples and vehicle samples.

To further characterize the safety of silvestrol, possible mutagenic and genotoxic effects were assessed. To determine the mutagenic potential of silvestrol, an Ames test was performed. Silvestrol was tested on the *Salmonella typhimurium* strains T100 and T98. These two strains carry a defective gene that renders them unable to synthesize the amino acid histidine. Drugs that induce back mutations that cause the gene to regain its function result in bacterial growth in a histidine-free medium (for details, see Materials and Methods section). Silvestrol was also incubated with T100 and T98 in the presence of the liver homogenate S9 to simulate its putative metabolic conversion by liver enzymes. Under all conditions, silvestrol was negative in Ames mutagenic assays (Figure 2A).

The genotoxic potential was analyzed with the micronuclei assay in HepG2 cells. The assay allows for the determination of micronuclei, indicating double-strand breaks in DNA and hypodiploids as indicators of chromosome loss and, in this way, clastogenic and aneugenic compounds can be identified [18]. The OECD guideline 487 states that several criteria need to be fulfilled to consider a compound as genotoxic, including a statistically significant and dose-related increased frequency of micronuclei, as well as hypodiploidy, with results outside the range of negative control data [18]. The positive control colchicine fulfilled all criteria, whereas the negative control ibuprofen showed no effects (Figure S2). We observed that at higher concentrations (50 nM), silvestrol mediates clastogenic effects in HepG2 cells, whereas no aneugenic effects were observable (Figure 2B). 

To investigate whether this genotoxic profile is based on the inhibition of elF4A by rocaglates, the elF4A inhibitor zotatifin (currently undergoing two clinical trials) was also tested. Only at lower concentrations (10 nM), zotatifin revealed some clastogenic and aneugenic effects (Figure 2B), but importantly, the effects were not concentration dependent. These data show that inhibition of elF4A with various rocaglates results in different genotoxic profiles.

### 2.3. Silvestrol Has No Effects on Ca^2+^, cAMP or IP_3_ Levels

**Figure 3:**
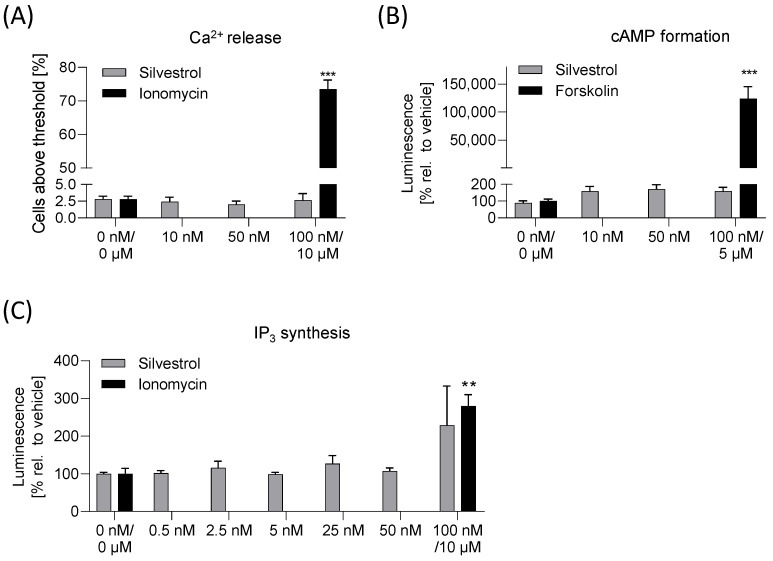
No GPCR-mediated off-target effects of silvestrol. (**A**) For the calcium assay, HEK293T cells were preincubated with Fluo-8-AM. Increasing concentrations of silvestrol, vehicle or 10 µM ionomycin (positive control) were added for 5 min. Data shown are the percentages of cells above the threshold. (**B**) For the cAMP assay, HEK293T cells were transfected with a GloSensor™ luciferase that in presence of cAMP lead to a conformational shift which in turn leads to an activation of the luciferase. The transfected cells were treated with increasing concentrations of silvestrol, vehicle or with 5 µM forskolin (positive control). The silvestrol and forskolin-treated samples were normalized to the respective vehicle control. (**C**) For the IP_3_ assay, HEK293T cells transfected with pGloSensor-22F cAMP plasmid were treated with silvestrol or 10 µM ionomycin (positive control). The chemiluminescence signal was detected with a spectrophotometer. The silvestrol- and ionomycin-treated samples were normalized to the respective vehicle control. The experiments were performed in biological triplicates. Data are expressed as mean ± SEM. For statistical analysis of the positive controls an unpaired *t*-test was used. ** *p* < 0.01, *** *p* < 0.001 indicate significant differences between positive controls and vehicle samples.

Side effects may be mediated via G-protein-coupled receptors (GPCRs), whose main function is to transduce extracellular signals and regulate intracellular second messengers, such as cyclic adenosine monophosphate (cAMP), Ca^2+^ and inositol 1,4,5-triphosphate (IP_3_), through coupling to heterotrimeric G proteins and their downstream effectors. Proliferation of cells is often regulated via GPCRs; thus, anti-tumor effects of silvestrol could be linked to factors involved in such signaling cascades. To identify potential off-target effects of silvestrol, Ca^2+^-release, cAMP and IP_3_ assays were performed. As expected, the positive controls, ionomycin or forskolin, induced significant increases in Ca^2+^-release, cAMP formation or IP_3_ synthesis. Importantly, silvestrol did not significantly induce Ca^2+^-release (Figure 3A), cAMP (Figure 3B) or IP_3_ synthesis (Figure 3C), demonstrating that silvestrol does not mediate off-target effects via GPCRs.

### 2.4. Silvestrol Is Stable in Human Liver Microsomes

**Figure 4:**
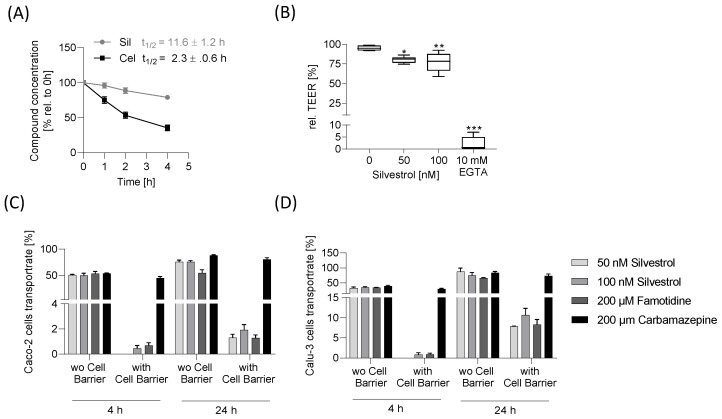
Stability and permeability of silvestrol. (**A**) Stability of silvestrol in human liver microsomes. 100 nM silvestrol and 10 µM celecoxib were incubated for different time periods with human liver microsomes. The concentrations of silvestrol and celecoxib were determined by LC-MS/MS and presented as the remaining percentage over time. The half-life was calculated by plotting the log of the test compound concentration versus incubation time and the slope of the initial linear portion of the curve was determined and used with the equation given in the Methods section. The experiment was performed in biological triplicates. Data are expressed as mean ± SEM. (**B**) For the influence of silvestrol on Caco-2 cell barrier integrity, the TEER values as a function of time in the presence or absence of different silvestrol concentrations (0, 50, 100 nM) or of 10 mM EGTA (positive control) was detected with a cellZscope2 device. To obtain the relative TEER values, TEER values 20 h after adding silvestrol were correlated to the values obtained shortly before adding silvestrol. The experiment was conducted in three biological triplicates. Data are expressed as mean ± SEM. For statistical analysis ordinary one-way ANOVA with Dunnett’s multiple comparisons test was used. * *p* < 0.05, ** *p* < 0.01, *** *p* < 0.001 indicate significant differences between compound samples and vehicle samples. (C/D) To determine the transport rate of silvestrol, Caco-2 (**C**) or Calu-3 (**D**) cell barriers were incubated with various concentrations of silvestrol, 200 µM carbamazepine or 200 µM famotidine for 4 h or 24 h. Transwell filters without cell barriers served as control. The concentration of the compounds in the apical and basolateral medium were determined using LC-MS/MS. The transport rate was calculated with equation given in the Methods section.

Next, we investigated, in vitro, the intrinsic clearance of silvestrol. For this purpose, we used human liver microsomes that contain membrane-bound drug-metabolizing enzymes. We selected concentrations for silvestrol (100 nM) and celecoxib (10 µM) that cover the respective IC_50_ or EC_50_ values (celecoxib: IC_50_ ≈ 1 µM for inhibition of COX-2 [19]; silvestrol: EC_50_ ≈ 1–100 nM for inhibition of virus replication) [20]). Silvestrol (100 nM) and the reference drug celecoxib (10 µM) were incubated for various time periods with human liver microsomes and the concentrations of the compounds were determined by LC-MS/MS. For celecoxib, a half-life of 2.3 ± 0.5 h and an intrinsic clearance of 10.5 ± 2 µL/min mg protein were detected, which fits partially with published data reporting an intrinsic clearance of 23 ± 5 µL/min mg protein [21]. For silvestrol, a half-life of 11.6 ± 1.2 h and an intrinsic clearance of 1.7 ± 0.5 µL/min mg protein were calculated, indicating that silvestrol is relatively stable in the presence of metabolizing enzymes (Figure 4A).

### 2.5. No Disturbance of Cell Barrier Integrity by Silvestrol

The epithelial cell layers serve as functional barriers in multicellular organisms. The junctions between adjacent cells are key components in epithelial cell barriers. Particularly relevant for the active barrier function of the cell layer are tight junctions, because they regulate the transport of molecules across the barrier by selectively opening and closing in response to various signals from outside and inside the cells. Moreover, efflux pumps, such as the P-glycoprotein, influence the transport rate of compounds [22]. To enter tissues, drugs have to pass through these tissue barriers. Between the permeability of a cell layer and its electrical resistance (measured as the transepithelial electric resistance (TEER)), a direct correlation exists. Therefore, to quantify the tightness of the barrier, the TEER value can be used. To characterize whether silvestrol influences the integrity of the cell barrier, the Caco-2 cell barrier was generated on a porous membrane and TEER values were recorded. EGTA served as a positive control because it depletes extracellular Ca^2+^ and leads, thereby, to a disassembly of tight junctions [23]. As expected, EGTA caused a drop in TEER readings (Figure 4B), whereas silvestrol reduced TEER readings only slightly (Figure 4B).

### 2.6. Low Transport Rate of Silvestrol in the Caco-2 and Calu-3 Cell Barrier Assay

To assess its permeability, the amount of silvestrol that passes through a Caco-2 cell barrier was detected. The compounds carbamazepine and famotidine were used as high- and low-permeability controls according to FDA guidelines for bioavailability (FDA, 2017). A Caco-2 cell barrier generated on a porous membrane was covered with silvestrol (50 nM, 100 nM), 200 µM carbamazepine or 200 µM famotidine for 4 and 24 h. Porous membranes without a cell barrier, incubated with different compounds, served as control. As expected, the transport rates of compounds obtained from samples without a cell barrier were over 50% (Figure 4C). The transport rate of carbamazepine after 4 h was approx. 45% and increased with time. The transport rate of famotidine was 0.7% after 4 h and increased to 1.3% after 24 h. For 50 nM silvestrol, only a transport rate of 1.3% was detectable after 24 h. At 100 nM, silvestrol has transport rates of 0.5% and 1.9% after 4 and 24 h, respectively (Figure 4C). These data indicate that silvestrol transport rate is within a similar range compared to that of famotidine and exhibits low permeability across Caco-2 cell barriers.

In addition to oral treatment, inhalation could also be a possible administration route; therefore, we investigated permeability in a cell barrier composed of airway-derived Calu-3 cells. Carbamazepine showed a transport rate of 74% and famotidine of 8.3% after 24 h, indicating that the Calu-3 cell barrier is more porous than the Caco-2 cell barrier. This assumption was further confirmed by the TEER values that were about four-times lower in the Calu-3 cell barrier (≈300 Ω cm^2^) than in the Caco-2 cell barrier (≈1200 Ω cm^2^) at the beginning of the transport assay. Silvestrol at a concentration of 50 nM was only detectable after 24 h (TR: 7.8%) and at 100 nM, silvestrol revealed transport rates of 0.8% and 10.6% over 4 or 24 h (Figure 4D). These data show that silvestrol exhibits higher permeability in the Calu-3 cell barrier than in the Caco-2 cell barrier, classifying this compound within a similar range to that of famotidine. Our data indicate that administration via inhalation could be the preferred administration route for silvestrol, especially when respiratory viruses will be treated.

### 2.7. Silvestrol Is Absorbed by Cells

**Figure 5:**
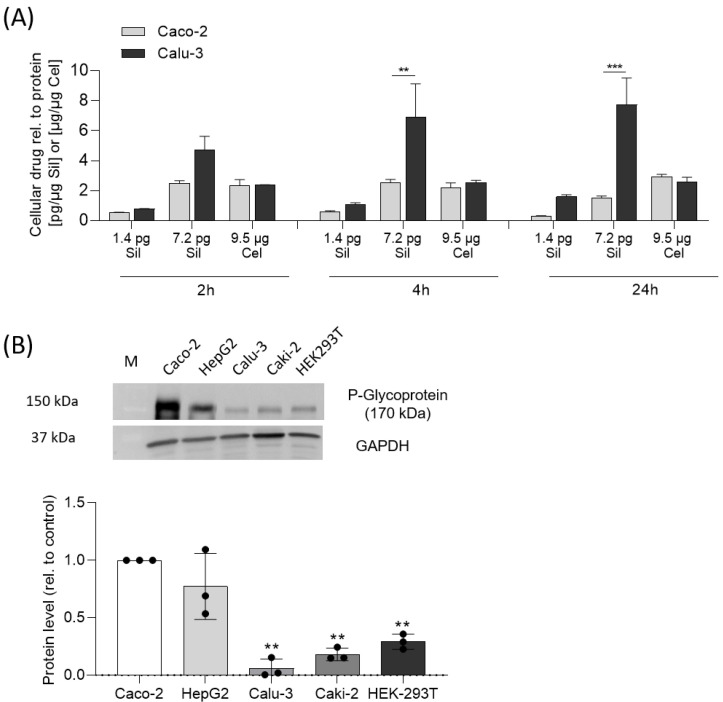
Cellular uptake assay. (**A**) Caco-2 or Calu-3 cells were incubated with two concentrations of silvestrol (5 nM, 25 nM) or 25 µM celecoxib (positive control) for the indicated time periods. The concentrations of the compounds were determined by LC-MS/MS. The concentration of drugs in the cells was related to the protein concentration. The experiments were performed in biological triplicates. Data are expressed as mean ± SEM. For statistical analysis, two-way ANOVA with Sidak’s multiple comparisons test was used. *** *p* < 0.001 indicate significant differences between Caco-2 and Calu-3 samples. (**B**) P-glycoprotein level was detected by Western blot and analyzed by the optical densitometric analysis achieved with the Image Lab software version 6.0 (Bio-Rad Laboratories, Hercules, CA, USA). The molecular weight of P-glycoprotein is 170 kDA including 10–25 kDa of N-terminal glycosylation. The protein level was normalized to GAPDH and related to the P-glycoprotein level of Caco-2 cells. Data are expressed as mean ± SEM. For statistical analysis, the relative P-glycoprotein level of the various cell lines was compared to the expression in Caco-2 cells using one-way ANOVA with Dunnett’s multiple comparisons test. ** *p* < 0.01 indicate significant differences.

Next, we investigated the cellular uptake of silvestrol in Caco-2 and Calu-3 cells. The approved drug celecoxib, which accumulates in cells [24] and is characterized by a high distribution volume [25], served as a positive control. The concentrations of silvestrol and celecoxib were determined by LC-MS/MS. A such, 25 µM celecoxib showed an uptake rate of about 2.5 ng/µg protein (≈2.3% celecoxib uptake related to used celecoxib), independent of time or cell type (Figure 5A). For Caco-2 cells, we found a concentration-dependent uptake of silvestrol with a maximum of about 2.5 pg/µg protein (25 nM silvestrol) over 2 and 4 h (≈1.3% silvestrol uptake related to used silvestrol). For Calu-3 cells, concentration- and time-dependent uptake of silvestrol, with a maximum of about 7.7 pg/µg protein, was observed (≈3.7% silvestrol uptake related to used amount of silvestrol) (Figure 5A). These data indicate that the cell-type-dependent uptake of silvestrol is within the same range as that of celecoxib.

### 2.8. Cytotoxicity of Silvestrol Depends on P-glycoprotein Level

Silvestrol is a substrate of the ATP-dependent efflux pump P-glycoprotein [26], the expression level of which is higher in Caco-2 than that in Calu-3 cells [27]. We hypothesized that in cells with a high P-glycoprotein level, silvestrol is ejected from cells, resulting in reduced intracellular silvestrol concentration and lower cytotoxicity. To evaluate this hypothesis, we investigated the P-glycoprotein level in the relevant cell lines (Caco-2, Calu-3, HepG2, HEK293T, Caki-2). Interestingly, the highest P-glycoprotein level was found in cell lines (Caco-2 and HepG2) responding with low cytotoxicity towards silvestrol, whereas in cells (Calu-3, HEK293T and Caki-2) responding to silvestrol with high cytotoxicity, the P-glycoprotein level was significantly lower (Figure 5B).

**Figure 6:**
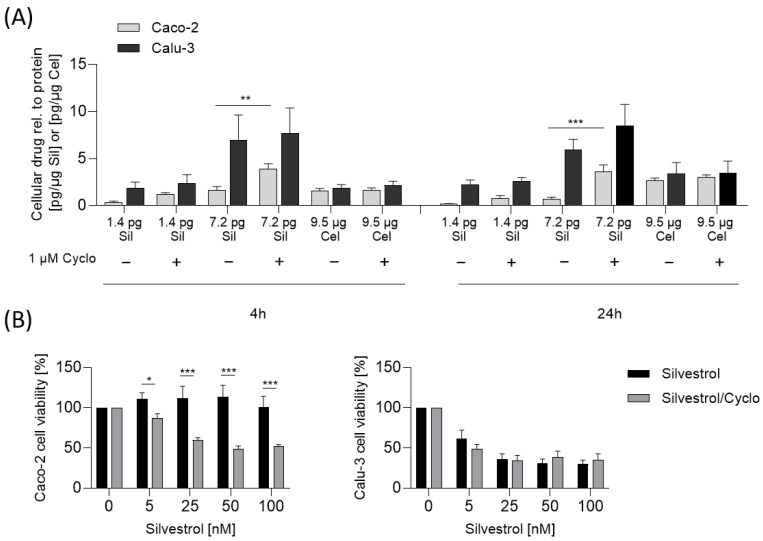
Cytotoxicity and cellular uptake of silvestrol vary in relation to P-glycoprotein levels. (**A**) For cellular uptake assay, Caco-2 or Calu-3 cells were preincubated with 1 µM cyclosporine to inhibit P-glycoprotein or vehicle for 60 min and subsequently incubated with various concentrations of silvestrol (5 nM, 25 nM) or 25 µM celecoxib for the indicated time points. The concentrations of the compounds were detected by LC-MS/MS. The concentration of drugs in the cells were normalized to the protein concentration. (**B**) For cytotoxicity, Caco-2 and Calu-3 cells were preincubated with 1 µM cyclosporine or left untreated for 60 min and subsequently incubated with increasing concentrations of silvestrol as indicated for 48 h. The viability was assessed with a formazan-based proliferation assay. The relative viability values were obtained by normalizing the values of the drug-treated samples to the control samples. The experiments were performed in biological triplicates. Data are expressed as mean ± SEM. Two-way ANOVA with Sidak’s multiple comparisons test was used for statistical analysis. * *p* < 0.05, ** *p* < 0.01, *** *p* < 0.001 indicate significant differences between compound and control samples (**A**) or Caco-2 and Calu-3 samples (**B**). Abb.: Cyclo, Cyclosporine.

We further investigated whether the inhibition of the P-glycoprotein by cyclosporine increases the intracellular concentration of silvestrol and sensitizes Caco-2 cells to silvestrol. Indeed, the inhibition of P-glycoprotein leads to an increase in intracellular silvestrol levels in Caco-2 cells, but not in Calu-3 cells (Figure 6A). As expected, the inhibition of P-glycoprotein increased the cytotoxic potential of silvestrol in Caco-2 but not in Calu-3 cells (Figure 6B). Next, we tested whether the synthetic rocaglate zotatifin is also a substrate of P-glycoprotein. As expected, the inhibition of P-glycoprotein increases the cytotoxicity of zotatifin in Caco-2 but not in Calu-3 cells (Figure S3), indicating that zotatifin is also a substrate of P-glycoprotein. Moreover, these data indicate that the cytotoxicity of silvestrol and zotatifin depends on the accumulation of these rocaglates in cancer cell lines due to low P-glycoprotein level.

## 3. Discussion

Silvestrol, originally described as a potent anti-tumor compound [28], was recently identified as a promising antiviral drug candidate with broad-spectrum activity [3,6]. Initially, the antiviral activity of silvestrol was identified in primary human macrophages infected with the Zaire Ebolavirus (ZEBOV) [3]. Importantly, mRNAs from EBOV are translated in a cap-dependent manner and harbor well-defined stable RNA secondary structures in their 5’-untranslated regions that need to be unwound to allow the binding of the 43S–preinitiation complex [29]. Therefore, it was not unexpected that EBOV requires eIF4A for translation initiation and, consequently, for its replication [3]. Usually, the next step on the path towards drug development includes prediction of potential bioavailability and an in vitro safety profile. Here, we performed relevant preclinical experiments in order to characterize the potential of silvestrol as an antiviral drug candidate in more detail. Our data indicate that silvestrol did not impair Caco-2 cell barrier integrity and has low permeability through Caco-2 and Calu-3 cell barriers. Silvestrol shows good stability in liver microsomes and good cellular uptake in cells expressing low levels of P-glycoprotein. Furthermore, we observed no off-target effects of silvestrol regarding GPCR signaling pathways, no mutagenic potential (Ames test) and a low genotoxic potential at higher concentrations. This apparently minor genotoxicity will need to be confirmed and characterized in further in vitro and in vivo studies. Regarding its cytotoxicity, silvestrol reduced cell viability in kidney cell lines HEK293T and Caki-2 with a CC_50_ of 16 and 37 nM, respectively, whereas in Caco-2, HepG2 and Calu-3 cells, concentrations up to 1000 nM silvestrol reduce viability only by about 50%. In other studies, cell-type-dependent cytotoxicity with silvestrol has also been observed. For example, in the lung cancer cell line A549, the CC_50_ value was 9.42 nM [5] and in the colon cancer cell line HT-29, the CC_50_ value was 0.7 nM [17]. Importantly, in several non-transformed primary cells, silvestrol has no major cytotoxicity [6,20,30].

To estimate transport across the intestinal or lung epithelium, the Caco-2 and Calu-3 cell barrier model was used. Silvestrol showed a low transport rate in both barrier models, indicating low permeability. Our in vitro data, thereby, confirm the results of a previous pharmacokinetic study in mice, which revealed that the bioavailability of silvestrol depends on the administration route. In this earlier study, mice were treated with silvestrol formulated in hydroxypropyl-β-cyclodextrin via intravenous, intraperitoneal and oral routes. Intraperitoneal systemic availability was 100%, but oral administration resulted in only 1.7% bioavailability [30]. The low oral bioavailability and the observed potential cytotoxic effects suggest that silvestrol would best be administered locally for treatment of infections caused by respiratory viruses via inhalation using a targeted aerosol formulation. This would have the advantage that the poor absorption through the intestine, transport via the bloodstream and, thus, cytotoxic effects on peripheral immune cells can all be avoided. Improvement in silvestrol’s bioavailability without affecting its antimicrobial efficacy by suitable formulations, such as solid dispersions [31], may also be an option to increase the local concentration of silvestrol in the infected respiratory tract.

Surprisingly, the high level of P-glycoprotein in Caco-2 cells did not result in a higher transportation rate of silvestrol. In a previous study, it was shown that a Caco-2 cell monolayer exhibits apical efflux of drugs, indicating that substrates of P-glycoprotein are transported in the reverse direction back to the apical compartment [22]. Therefore, instead of using the transcellular route to the basolateral compartment, P-glycoprotein may shuttle silvestrol back to the apical compartment.

As mentioned, the concentration of silvestrol was higher in the lung cancer cell line Calu-3 than in the colon cancer cell line Caco-2. One explanation for the observed cell-type-dependent cytotoxicity could be that the cellular uptake and/or the efflux of silvestrol differs in these cell types due to different cellular P-glycoprotein levels. Calu-3 cells express lower levels of P-glycoprotein in comparison to Caco-2 cells, as shown by Hamilton et al. [27] and confirmed in our experiments (see Figure 5B). We assumed that the cell-type-dependent, intracellular silvestrol level depends on an increased efflux of silvestrol and not on different absorption rates. This would also fit with the observed time-dependent increase in uptake into Calu-3 compared to Caco-2 cells. In cells with increased efflux of silvestrol, the equilibrium between uptake and efflux is reached earlier and, consequently, no further increase in cellular silvestrol concentration can be observed. Moreover, celecoxib, which is not a substrate of P-glycoprotein [32], showed similar uptake rates in Caco-2 and Calu-3 cells. Accordingly, the cytotoxicity of silvestrol correlates with the level of P-glycoprotein in cancer cell lines. In line with this, inhibition of P-glycoprotein by cyclosporine increased the intracellular silvestrol levels and, therefore, the sensitivity of Caco-2 cells towards the cytotoxicity of silvestrol. These data indicate that the cell-type-dependent cytotoxicity of silvestrol depends on its intracellular concentration, which is determined not only by cellular uptake but also by its efflux from the cell.

The CC_50_ value of silvestrol increased in primary monocytes (CC_50_ = 29 nM), M1 macrophages (CC_50_ = 45.6 nM) and M2 macrophages (CC_50_ > 100 nM), in that order [9]. Interestingly, the P-glycoprotein level in U937 monocytes, M1 macrophages and M2 macrophages increased in the same order [33]. These data indicate that P-glycoprotein level in primary monocytes, M1 and M2 macrophages could also be responsible for the different CC_50_ values of silvestrol in these cell types. For T cells expressing high levels of P-glycoprotein, we found no cytotoxic effects up to 100 nM silvestrol [9,34]. Moreover, the genotoxic effects observed in HepG2 cells may also be caused by high intracellular silvestrol concentration, since HepG2 cells have about 10-fold lower P-glycoprotein levels compared to Caco-2 cells [35]. Given the wide distribution of P-glycoprotein in the body [36] and in immune cells (e.g., B cells, T cells, natural killer cells) [34,37], its relevance for the effects of silvestrol in vivo will require careful consideration.

The cytotoxicity of a drug can be considered tolerable if the CC_50_ is at least 50-fold higher than the effective concentration (EC_50_) [16]. In our study, we observed a cell-type-dependent cytotoxic effect, with the lowest CC_50_ value of 16 nM in HEK293T cells. However, cytotoxicity in cancer cells, but not in healthy cells, is a prerequisite for an anti-cancer compound like silvestrol. Of course, in several primary cells, the selectivity index (SI) of silvestrol (SI calculated by CC_50_/EC_50_), regarding its antiviral activity, can reach 1000 and virus titers can be reduced by up to 4 log phases [5]. Therefore, silvestrol is a very promising candidate for further antiviral drug development. In accordance with this, the synthetic rocaglate zotatifin has already reached the clinic in a dose-escalation study in patients with mild or moderate COVID-19 [38].

Like silvestrol, zotatifin also induces a G2 cell cycle block and apoptosis at 100 nM in the MDA-MB-231 cell line [39], indicating that the inhibition of the target elF4A mediates cytotoxic effects. However, in primary immune cells, zotatifin mediates lower cytotoxicity in comparison to silvestrol, whereas CR31B (-), another synthetic rocaglate, has comparable cytotoxic effects to those of silvestrol [9]. Since rocaglates are highly specific eIF4A inhibitors, our data indicate that the inhibition of elF4A is mainly responsible for the observed cell-type-dependent cytotoxic effects. However, additional secondary effects based on reduced translation of cellular mRNAs should also be considered. It can be expected that different rocaglates have different effects on the pool of mRNAs in a given cell. For example, only the natural compound silvestrol contains a dioxane moiety that could promote polypurine-independent RNA-clamping, whereas synthetic rocaglates without the dioxane moiety need a polypurine stretch to clamp the RNA substrate efficiently onto the surface of eIF4A [5].

Interestingly, silvestrol reduces energy metabolism [40] and cancer cell lines have a higher energy requirement than primary cells due their higher proliferation rate [41]. In line with this, several proto-oncogenes have complex 5’-UTRs with stable RNA secondary structures that need to be unwound by eIF4A during translation initiation. For example, eIF4A-dependent mRNAs, including critical Kirsten rat sarcoma virus (KRAS) signaling molecules, such as phosphoinositide 3-kinase (PI3K), Ras-related protein (RalA), Ras-related C3 botulinum toxin substrate 2 (Rac2) and MYC, were identified in pancreatic adenocarcinoma by transcriptome-scale ribosome footprinting [42]. These data further indicate that the effects of rocaglates on proliferation could be mediated by the translation inhibition of proto-oncogenic mRNAs, which drive the cell cycle. Nevertheless, more extensive global analyses of rocaglate-treated primary cells by proteomics and/or ribosome profiling approaches should be performed to gain further information on eIF4A-dependent mRNAs in infection-relevant cell systems.

The main therapeutic indicator from the studies discussed is that bioavailability of silvestrol is rather low, indicating that oral application should be avoided and local administration, for example, via inhalation, should be clearly preferred. A further important finding of our study is that silvestrol shows minor genotoxicity at higher concentrations. These potential side effect should be addressed in further studies. The cell-type-dependent effect could be relevant for side effects in cancer and infection treatment, since cells with high P-glycoprotein expression are potentially protected from side effects, but possibly also excluded from treatment effects.

Taken together, our findings indicate that silvestrol, due to its cell-type-dependent cytotoxicity and low permeability, should be administered directly into the respiratory tract at least to combat respiratory viruses.

## 4. Materials and Methods

### 4.1. Cells and Reagents

Caki-2 cells were cultured in McCoy’s 5A (modified) medium supplemented with 10% FCS, Calu-3 cells were cultured in MEM with 10% FCS and 1x non-essential amino acids and both obtained from ATCC (Manassas, VA, USA). HEK293T cells were cultured in DMEM supplemented with GlutaMAX, 10% heat-inactivated FCS. Caco-2 cells were from Sigma Aldrich (Schnelldorf, Germany) and were cultured in EMEM medium supplemented with 10% FCS, L-glutamine and non-essential amino acids (M7145, Sigma Aldrich, Schnelldorf, Germany). HepG2 was obtained from Sigma Aldrich (Schnelldorf, Germany) and cultured in DMEM supplemented with GlutaMAX and 10% heat-inactivated FCS. All media contained 1% penicillin/streptomycin and the cells were cultured at 37 °C in a 5% CO_2_ atmosphere. Silvestrol was dissolved in DMSO and further diluted in media (c_stock_ = 6 mM, maximal DMSO concentration during experiments 0.1% *v*/*v*). EGTA, famotidine and carbamazepine were from Sigma Aldrich (Schnellendorf, Germany). Lucifer Yellow (sc-215269) was obtained from Santa Cruz. Celecoxib was synthesized by WITEGA Laboratorien Berlin-Adlershof GmbH (Berlin, Germany). Forskolin and ionomycin were obtained from Sigma Aldrich (Schnelldorf, Germany). Silvestrol was provided by the Sarawak Biodiversity Centre (Kuching, North-Borneo, Malaysia; purity > 99%) and zotatifin was bought from MedChemExpress (USA, purity: 98%).

### 4.2. Cell Viability/Proliferation Assays

The Orangu^TM^ kit for viability testing was performed as described by the supplier. Briefly, 2.0 × 10^4^ Caco-2 cells, 2.0 × 10^4^ Calu-3 cells, 2.0 × 10^4^ HEK293T cells or 1.0 × 10^1^ Caki-2 cells were incubated for 24 h at 37 °C. Silvestrol (0–500 nM or 0–5000 nM (Caco-2 cells)) and control (DMSO) were added to the cells and mixed. After 48 h, 10 µL Orangu (WST-8) reagent (Cell Guidance System, Cambridge, UK) was added, mixed and incubated at 37 °C for 60 min. An EnSpire plate reader (PerkinElmer, Waltham, MA, USA) was used to measure absorbance at 450 nm and at 650 nm (reference). The absorbance at 450 nm was normalized with the absorbance at 650 nm. The sample values were corrected with the background wells (wells with medium and without cells). The absorbance of DMSO-treated cells was set to 100% and the silvestrol samples were correlated to the absorbance value of DMSO-treated samples to calculate cell viability.

### 4.3. Ames Test

The Ames MPF 98/100 test (Xenometrix, Allschwil, Switzerland) was performed as recommended by the supplier and recently published [43]. Briefly, two *Salmonella typhimurium* strains TA98 and TA100 (incapable to produce histidine) were used with or without liver S9 homogenate to simulate the metabolic conversions of silvestrol with liver enzymes. After exposure to increasing concentrations of silvestrol or positive controls (2 μg/mL of 2-NF (TA98), 0.1 μg/mL of 4-NQO (TA100), 2.5 μg/mL (TA100) and 1.0 µg/mL (TA98) of 2-AA), the cultures were diluted in pH indicator medium lacking histidine and incubated for two days. Cells that had undergone reversion to amino acid prototrophy grew into colonies and bacterial metabolism reduces the pH of the medium, changing the color of that well. The number of wells containing revertant colonies were counted and compared to a negative control (DMSO). The experiment was performed once in triplicate. The Ames MPF calculation sheet provided by Xenometrix (Allschwil, Switzerland) was used for data analysis. Fold induction over the baseline was taken as the ratio of the mean number of positive wells for the dose concentration divided by the baseline. The baseline was obtained by adding one standard deviation to the mean number of positive wells of the solvent control. Compounds inducing revertant numbers above the baseline were characterized as substances with mutagenic potential.

### 4.4. Micronuclei Assay

The In Vitro Micronuclei Plus assay (Becton Dickinson, Heidelberg, Germany) was performed as described by the supplier. The assay was performed with HepG2 cell line since Valentin-Severin et al. identified this cell line as a suitable tool to study genotoxicity [44]. Briefly, 2.0 × 10^4^ HepG2 cells were seeded for 24 h and incubated with increasing concentrations of silvestrol and zotatifin for 4 h. After a washing step with PBS, fresh medium (without test substance) was added and cells were incubated for 44 h. The assay was stopped by placing the plate on ice for 20 min and the medium was carefully removed, taking care not to disturb the cell surface. Further, 50 µL of Complete Nucleic Acid Dye A Solution was added and the plate was placed on ice and exposed to visible light for 30 min. The Nucleic Acid Dye A solution was removed and the cells were washed with 0.15 mL of cold 1x Buffer Solution. Then, 100 µL Complete Lysis Solution 1 was added, followed by gently mixing the plate for 5 s and incubation of 1 h in the dark at 37 °C. After this, 100 µL Complete Lysis Solution 2 was added and incubated for 30 min in the dark at room temperature. The cells were analyzed by flow cytometry with the MACSQuant 10 (Miltenyi GmbH, Bergisch Gladbach, Germany).

### 4.5. Analysis of Intracellular Ca^2+^ Levels

Next, 50,000 HEK293T cells were seeded in a 96-well poly-D-lysine-coated plate and incubated at 37 °C for 24 h. Cells were incubated with 4 µM Fluo-8-AM in HBSS for 1 h at 37 °C. After 1 h, the Fluo-8/HBSS was replaced by 100 µL HBSS. Five images per second were taken using an ImageXpress Micro Confocal High Content Imaging System (Molecular Device, San Jose, CA, USA). Silvestrol (10 nM, 50 nM, 100 nM), 5 µM ionomycin (Sigma Aldrich; positive control) or DMSO (negative control) was added to the cells. An image was taken every second for the next 20 s. The MetaXpress Software Version 6.7.1.157 (San Jose, CA, USA) was used to analyze the data. A threshold of fluorescence intensity was defined using cells before treatment and all cells with a fluorescence signal above the threshold level were counted and related to all cells.

### 4.6. Analysis of cAMP Synthesis

The cAMP assay was performed as recommended by the supplier. HEK293T cells were transfected with pGloSensor-22F cAMP plasmid (Promega, Walldorf, Germany) using the turbofect reagent (Thermo Fisher Scientific, Dreieich, Germany). As such, 8.0 × 10^4^ of transfected cells were seeded on a 96-well poly-d-lysine plate and incubated for 24 h at 37 °C. Then, 100 µL DMEM supplemented with 2% pGlo Sensor cAMP Reagent Solution (Promega, Walldorf, Germany) was added. Background luminescence (relative light units) before addition of compounds was determined with the EnSpire plate reader (PerkinElmer, Waltham, MA, USA). To investigate whether silvestrol induces cAMP synthesis, silvestrol (0 nM, 10 nM, 50 nM, 100 nM) or 5 µM forskolin (positive control) was added and the luminescence was detected.

### 4.7. Stability Assay

To 0.5 mg/mL human liver microsomes (50 donor pool; GIBCO) diluted in 0.1 M potassium phosphate buffer (0.1 M K_2_HPO_4_, 0.1 M KH_2_PO_4_, ph 7.4), 100 nM silvestrol or 10 µM celecoxib was added. The reaction was started with 1 mM NADPH diluted in potassium phosphate buffer. As control, samples without NAPDH were used. After various time points (0 h, 1 h, 2 h, 4 h), the reaction was stopped by adding ice-cold acetonitrile. The samples were centrifuged (5 min, 4 °C, 10,000× *g*) and supernatant was stored at −80 °C until the remaining drug concentration was determined by LC-MS/MS as described in detail below. The concentrations of the drugs were plotted against time and the elimination constant (k) was calculated from the slope (m):

m = −k

Equation to determine half-life:

T_1/2_ = 0.693/K

Equation to determine intrinsic clearance:

Cl = Ln(2) × 1000/T_1/2_/protein concentration

### 4.8. Cell Barrier Model

The cell barrier model was set up as described previously [43]. Briefly, 2.0 × 10^4^ Caco-2 or 5.0 × 10^4^ Calu-3 cells in 300 µL medium were seeded on 24-well ThinCerts (Greiner Bio-One GmbH, Frickenhausen, Germany) (pre-coated with FCS for 30 min) and in the lower compartment, 1 mL culture medium per well was added. The Caco-2 cell barrier was generated over 20 days and the Calu-3 barrier was generated over 14 days. To the apical compartment, silvestrol (0 nM, 50 nM, 100 nM) diluted in medium was added. Further, 10 mM of EGTA served as control. The transepithelial electrical resistance (TEER) was documented for 24 h every 30 min. To calculate the relative TEER % value, TEER values at the time point at which the compound solution (t_0_ h) was added were related to the TEER value obtained after 20 h. For the transport assay, the Caco-2 and Calu-3 cell barrier was generated as mentioned above. The compounds carbamazepine and famotidine were used as high- and low-permeability controls according to FDA guidelines for bioavailability (FDA, 2017). Silvestrol (0 nM, 50 nM, 100 nM), 200 µM famotidine or 200 µM carbamazepine diluted in medium was added to the apical compartment and incubated for 4 h or 24 h. The basolateral and apical medium was collected and stored at −20 °C and analyzed by LC-MS/MS as detailed below.

### 4.9. Cellular Uptake Assay for Silvestrol and Celecoxib

The cellular uptake assay protocol of Bosnar et al. [45] was modified. Briefly, 2.0 × 10^5^ Caco-2 or Calu-3 cells per well were seeded in 24-well plates and incubated for 24 h. Cells were washed with 500 µL PBS, silvestrol (5 nM, 25 nM), celecoxib (25 µM) or vehicle diluted in medium, with 1 µM cyclosporine or without, were added and incubated for various time points (2 h, 4 h, 24 h). After incubation, medium was removed and cells were washed four times with 500 µL ice-cold PBS. Cells were harvested, centrifuged (300× *g*, 5 min, 4 °C) and medium was removed. Protein concentration was determined by Bradford assay (Sigma Aldrich, Schnelldorf, Germany). Silvestrol and celecoxib concentration in cells was determined by LC-MS/MS analysis as described below.

### 4.10. Determination of P-glycoprotein Abundance

Caco-2 cells, Caki-2 cells, HepG2 cells, Calu-3 cells and HEK293T cells were harvested and lysed in RIPA-buffer (25 mM Tris-HCl (pH7.6), 1% Sodium deoxycholate, 0.1% SDS, 1% IPEGAL, 150 mM NaCl, Roche cOmplete™ Mini tablets (Sigma Aldrich, Schnelldorf, Germany)). A bicinchoninic acid assay (Thermo Fisher Scientific, Schwerte, Germany) was used to assess protein concentrations.

Moreover, 100 µg (Caco-2 cells, Caki-2 cells, HepG2 cells, Calu-3 cells, HEK293T cells) of total protein extract was prepared for Western blot analysis without denaturation by heating, then separated electrophoretically by 8% SDS-PAGE and electroblotted onto nitrocellulose membranes (Amersham Life Science, Freiburg, Germany). EveryBlot Blocking Buffer (Bio-Rad Laboratories, Feldkirchen, Germany) was used to block membranes. All antibodies were diluted in TBS with 1% BSA and 0.1% Tween 20. Membranes were incubated with the respective P-glycoprotein (1:1000, overnight at 4 °C) and GAPDH (1:5000, 2 h at room temperature) primary antibodies, washed three times with 0.05% Tween 20 in TBS, incubated with an anti-rabbit AF488 or an anti-mouse AF546 antibody (1:10,000 each) in TBS with 1% BSA and 0.1% Tween 20 for 60 min and washed again three times with 0.05% Tween 20 in TBS. The ChemiDo^TM^ MP Imaging System from Bio-Rad Laboratories (Feldkirchen, Germany) was used to detect the fluorescence signals. The rabbit monoclonal anti-P-glycoprotein antibody and the mouse monoclonal anti-GAPDH were purchased from Abcam (Berlin, Germany) and the anti-rabbit AF488 and anti-mouse AF546 antibodies from Thermo Scientific (Schwerte, Germany).

### 4.11. Determination of Silvestrol, Carbamazepine, Famotidine and Celecoxib via LC-MS/MS

The concentrations of carbamazepine, silvestrol, famotidine and celecoxib were determined using three different LC-MS methods. Carbamazepine and silvestrol were determined using the same LC-MS method but with different sample pre-treatment protocols.

For the analysis of carbamazepine, 50 µL medium was mixed with 20 µL of the internal standard (IS) solution (carbamazepine-d10, 5 ng/mL in methanol) and 320 µL methanol. For calibration standards and quality control samples, 50 µL blank medium was spiked with 20 µL of standard working solutions and processed like the samples. After vortexing and centrifugation, 100 µL of the liquid phase was transferred to glass vials.

For the analysis of silvestrol, 50 µL medium was mixed with 20 µL of the IS solution (carbamazepine-d10, 5 ng/mL in methanol) and 250 µL methanol. For calibration standards and quality control samples, 50 µL blank supernatant was spiked with 50 µL of standard working solutions and processed like the samples. After vortexing and centrifugation, the liquid phase was removed and evaporated at a temperature of 45 °C under a gentle stream of nitrogen. The residues were reconstituted with 50 μL of methanol.

The LC-MS/MS analysis of silvestrol and carbamazepine was carried out using an Agilent 1290 Infinity LC system (Agilent, Waldbronn, Germany) coupled to a hybrid triple quadrupole linear ion trap mass spectrometer QTRAP 6500+ (Sciex, Darmstadt, Germany) equipped with a Turbo-V-source operating in positive electrospray ionization mode. The chromatographic separation was carried out using a Zorbax C8 Eclipse Plus RRHD column (50 × 2.1 mm, 1.8 µm particle size and 95 Å pore size; Agilent, Santa Clara, CA, USA), maintained at 40 °C (Figure S4). A gradient program was employed at a flow rate of 300 µL. Mobile phase A was 0.1% formic acid + 10 mM ammonium formate and mobile phase B was acetonitrile + 0.0025% formic acid.

For the analysis of famotidine, 40 µL sample was spiked with 20 µL methanol, 20 µL IS (famotidine-d4, 100 ng/mL in methanol), 280 µL H_2_O and 40 µL methanol. For calibration standards and quality control samples, 40 µL blank medium was spiked with 20 µL standard working solution, 20 µL IS, 280 µL H_2_O and 40 µL methanol. After mixing and centrifugation, the supernatant was transferred to glass vials. The analysis was performed with an Agilent 1200 Infinity LC system (Agilent, Waldbronn, Germany) coupled to a hybrid triple quadrupole linear ion trap mass spectrometer QTRAP 5500 (Sciex, Darmstadt, Germany) equipped with a Turbo-V-source operating in positive electrospray ionization mode. The autosampler was a CTC PAL (CTC Analytics AG, Zwingen, Switzerland). The chromatographic separation was carried out using a Synergi Hydro RP coloumn (150 × 2 mm, 4 µm; Phenomenex, Aschaffenburg, Deutschland), maintained at 50 °C. Mobile Phase A was 10 mM NH_4_Ac and mobile Phase B was acetonitrile.

For the analysis of celecoxib, 20 µL sample was spiked with 20 µL IS (celecoxib-d7, 50 ng/mL in ethanol) and 140 µL ethanol. For calibration curve and quality control samples, 20 µL standard working solution was spiked with 20 µL IS, 20 µL PBS and 120 µL ethanol. After mixing and centrifugation, the supernatant was transferred to glass vials. The analysis of celecoxib was performed with an Agilent 1200 Infinity LC system (Agilent, Waldbronn, Germany) coupled to a hybrid triple quadrupole linear ion trap mass spectrometer QTRAP 5500 (Sciex, Darmstadt, Germany) equipped with a Turbo-V-source operating in negative electrospray ionization mode. The autosampler was a CTC PAL (CTC Analytics AG, Zwingen, Switzerland). The chromatographic separation was carried out using a Synergi Max RP coloumn (150 × 2 mm, 4 µm; Phenomenex, Aschaffenburg, Germany), maintained at 35 °C. Mobile Phase A was water and mobile Phase B was acetonitrile. The LC method was an isocratic method, which started at 80% B for 6 min at 300 µL/min. Further, 10 µL sample was injected.

For analysis and quantification of all compounds, Analyst Software 1.7.1 and Multiquant Software 3.0.3 (both Sciex, Darmstadt, Germany) were used. The precursor-to-product ion transitions used for quantification were: *m*/*z* 237.1 → *m*/*z* 194.1 for carbamazepine, *m*/*z* 672.3 → *m*/*z* 535.1 for silvestrol, *m*/*z* 338.1 → *m*/*z* 259.1 for famotidine and *m*/*z* 380.1 → *m*/*z* 315.9 for celecoxib. Calibration curves were constructed using linear regression with 1/x weighting. Variations in accuracy were less than 15% over the whole range of calibration, except for the lowest limit of quantification, where a variation in accuracy of 20% was accepted.

### 4.12. Statistics

Results are presented as means ± standard errors (SEM). The data were analyzed with two-way or one-way analysis of variance (ANOVA) and with Dunnett’s or Sidak’s multiple comparisons test. For all calculations and creation of graphs, GraphPad Prism v8 was used and *p* < 0.05 was considered the threshold for significance. The concentration–response data were fitted to a 4-parameter logistic fit using GraphPad Prism v8 to yield the CC_50_ value.

## 5. Conclusions

Silvestrol, a natural rocaglate with potent anti-tumor and broad-spectrum anti-viral activity, was analysed regarding its in vitro safety, off-target and bioavailability profile. We found cell-type-dependent cytotoxicity of silvestrol dependent on cellular P-glycoprotein levels, which will need to be taken into consideration in clinical studies, as well as no mutagenic and only minor genotoxic potential. Putative off-target effects of silvestrol mediated by GPCR signaling could not be detected. Silvestrol shows high stability in human liver microsomes in the presence of metabolizing enzymes and good cellular uptake but low transport rates in colon-derived Caco-2 and lung-derived Calu-3 cell barrier assays. From our data we suggest the local administration of silvestrol by an aerosol to combat respiratory viruses based on its acceptable safety and bioavailability profile.

## Data Availability

Data are contained within the article and supplementary material.

## Supplementary Materials

The following supporting information can be downloaded at: https://www.mdpi.com/article/10.3390/ph15091086/s1, Figure S1: Impact of silvestrol on HepG2 cell viability; Figure S2: Micronuclei assay of ibuprofen and colchicin; Figure S3: Cytotoxic effects of zotatifin in Caco-2 and Calu-3 cells in dependency of inhibition of P-glycoprotein; Figure S4: Representative chromatograms of Silvestrol of silvestrol.
