# Supplementary material for: In Vitro Safety, Off-Target and Bioavailability Profile of the Antiviral Compound Silvestrol
Source: Pharmaceuticals (Basel). 2022 Aug 31;15(9):1086. doi: 10.3390/ph15091086 (PMC9502993; doi:10.3390/ph15091086)
Supplement: Supplementary file 1 [file pharmaceuticals-15-01086-s001.zip › pharmaceuticals-1837167-supplementary.pdf]

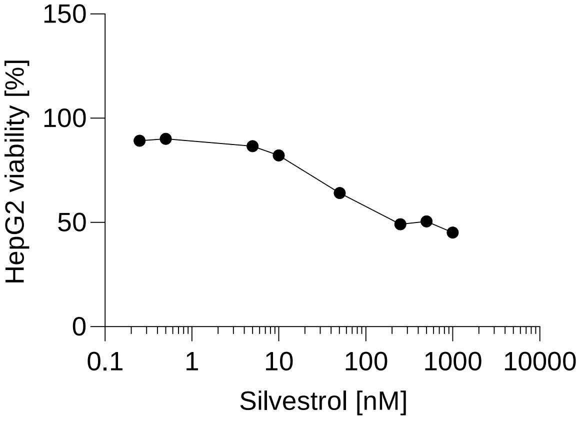

Figure S1: Impact of silvestrol on HepG2 cell viability. For the cell viability assay, HepG2 cells were incubated with various concentrations of silvestrol for 48h. The absorbance of DMSO-treated cells was set to 100%, and the silvestrol samples were normalized to the absorbance of the respective DMSO value to calculate cell viability. The experiments were performed in biological and technical triplicates. Data are expressed as mean ± SEM.

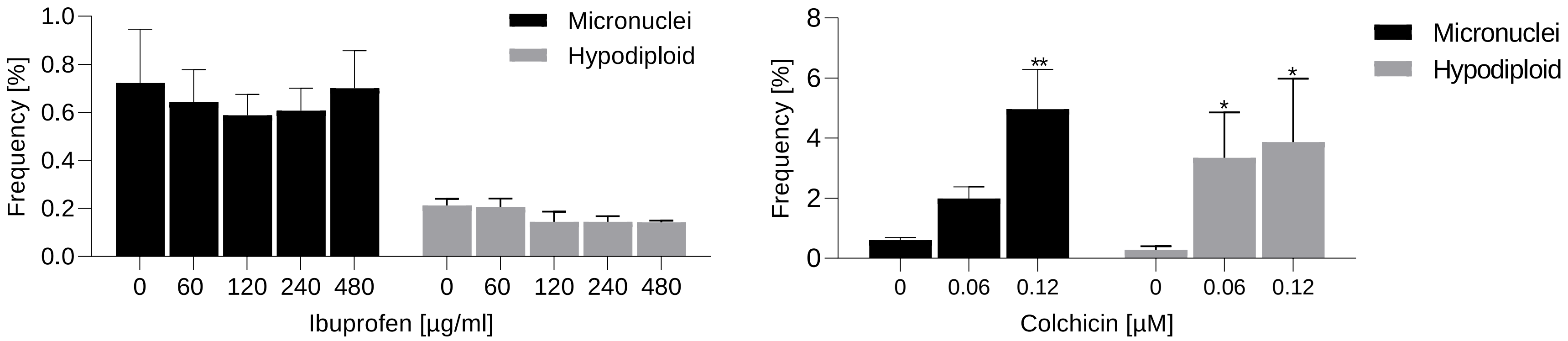

Figure S2: Micronuclei assay of ibuprofen and colchicine. For the micronuclei assay HepG2 cells were incubated with increasing concentrations of ibuprofen (negative control) and colchicine (positive control) or vehicle for 44 h. The analyses of micronuclei and hypodiploid cells were achieved by flow cytometry as recommended by the supplier of the in Vitro Micronuclei Plus assay. For statistical analysis, two-way ANOVA with Dunnett`s multiple comparisons test was used. *p<0.05, ** p<0.01 indicate significant differences between compound and control samples.

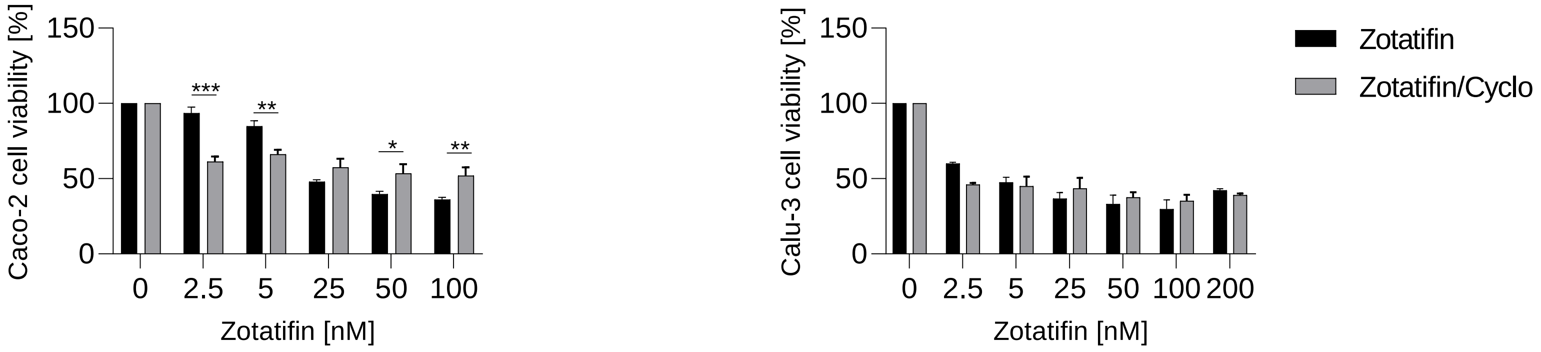

Figure S3: Cytotoxic effects of zotatifin in Caco-2 and Calu-3 cells with respect to inhibition of P-glycoprotein. Caco-2 and Calu-3 cells were preincubated with 1 µM cyclosporine or left untreated for 60 min subsequently the cells were incubated for 48h with increasing concentration of zotatifin as indicated. The viability was assessed with a formazan-based proliferation assay. The relative viability values were obtained by normalizing the values of the drug treated samples to the control samples. For statistical analysis, two-way ANOVA with Sidak`s multiple comparisons test was used. *p<0.05, ** p<0.01 indicate significant differences between compound and control samples. Abb.: Cyclo; Cyclosporine.

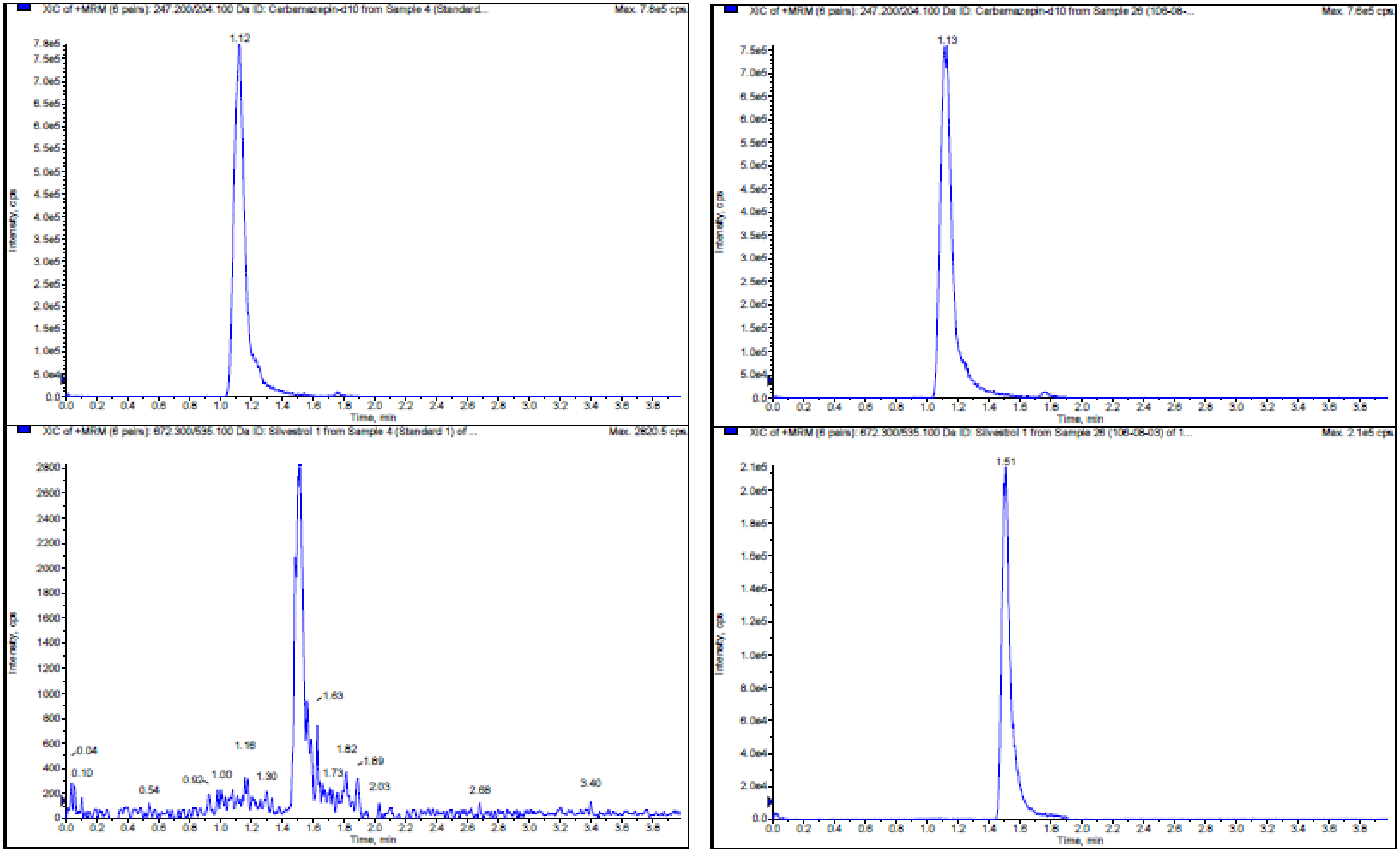

Figure S4: Representative chromatograms of Silvestrol (*m/z* 672.3 → 535.1, bottom row) and the internal standard Carbamazepine-d10 (*m/z* 247.2 → 204.1, upper row) at the lower limit of quantification (LLOQ, left side) and in one supernatant sample (right side).
